# The effect of *Ni* *gella sativa* and vitamin D3 supplementation on the clinical outcome in COVID-19 patients: A randomized controlled clinical trial

**DOI:** 10.3389/fphar.2022.1011522

**Published:** 2022-11-08

**Authors:** Shimaa A. Said, Alsayyed Abdulbaset, Amal A. El-Kholy, Osama Besckales, Nagwa A. Sabri

**Affiliations:** ^1^ Respiratory Specialized Hospital, Kobry Al Qobba Military Complex, Cairo, Egypt; ^2^ Department of Clinical Pharmacy, Faculty of Pharmacy, Ain Shams University, Cairo, Egypt

**Keywords:** *Nigella sativa*, vitamin D3, COVID-19, complementary medicine, immunomodulation, add-on therapy

## Abstract

**Background:** The coronavirus disease 2019 (COVID-19) is a novel coronavirus that causes severe infection in the respiratory system. Since the immune status plays an essential role in combating COVID-19, herbal medicines, which have an immunomodulatory effect, may help prevent and even treat COVID-19. *Nigella* *sativa* is one of the herbal medicines with antiviral and immunomodulatory activities, and its therapeutic effectiveness makes it a promising add-on therapy for COVID-19. In addition, vitamin D3 has an immunomodulatory role, but the effect of therapeutic vitamin D3 supplementation in SARS-CoV-2 infection is still not well-known.

**Objective:** This study aims to investigate the effects of *Nigella sativa* and vitamin D3 as single supplemental therapies and in combination on viral clearance indicated by a negative polymerase chain reaction and the alleviation of symptoms during the study follow-up duration of 14 days.

**Patients and Methods:** The study design was an open-label randomized controlled clinical trial conducted at the Respiratory Hospital at the Kobry El Qobba Armed Forces Medical Complex. In total, 120 COVID-19 patients with mild to moderate symptoms were randomly assigned to four groups, with thirty patients each, as follows: Group 1 received an oral dose of 900 mg *Nigella sativa* through 450 mg soft gelatin capsules twice daily for two weeks; Group 2 received 2,000 IU of vitamin D3 through 1000-IU tablets given as two tablets, once daily; Group 3 received 900 mg of *Nigella sativa* and 2,000 IU of vitamin D3 in the same manner of dosing as in the previous groups; and Group 4 was the control group. All groups received standard therapy for COVID-19 infections and clinical management of COVID-19’s clinical symptoms.

**Results:** The *Nigella sativa*–vitamin D3 combination in addition to the standard therapy for COVID-19 infections significantly contributed to the alleviation of most COVID-19 symptoms: 50% of patients were free of cough after 7 days, 70% showed an absence of fatigue after 4 days, 80% had no headache after 5 days, 90% were free of rhinorrhea after 7 days, and 86.7% of the patients had no dyspnea after 7 days. Moreover, patients in the four studied groups showed a reduced median temperature after 3 days of treatment. Negative results of the polymerase chain reaction (PCR) test recorded on the 7th and 14th day of therapy were superior in the *Nigella sativa* and vitamin D3 combination arm compared to those of the other studied arms where the value of the odds ratio (OR) on the 7th day was 0.13 with 95% CI: 0.03–0.45 and that of the 14th day was 0.09 with 95% CI: 0.02–0.3.

**Conclusion:** The results of this study showed a promising therapeutic benefit of the administration of *Nigella sativa* and vitamin D3 combination in COVID-19 patients with mild to moderate symptoms. Additionally, the remarkable viral clearance in a short time interval and reduction in the severity and progression of symptoms recommended the use of this combination as an add-on therapy for the management of COVID-19 patients.

**Clinical Trial Registration:** ClinicalTrials.gov, Identifier: NCT04981743.

## Introduction

Coronavirus disease 2019 (COVID-19) is caused by a novel coronavirus known as severe acute respiratory syndrome coronavirus 2 (SARS-CoV-2), which was recognized first in Wuhan City, China, and spread rapidly worldwide. As the illness spreads to new regions of the world, human understanding of its epidemiological characteristics is constantly changing; additionally, the full clinical presentation of COVID-19 is still not completely elucidated. It is well-known that the most common symptoms associated with COVID-19 are cough, fever, dyspnea, fatigue, anosmia, and headache, while some patients may suffer from a severe viral infection and weakened immune status with severe cytokine storms, pulmonary fibrosis, and multi-organ dysfunction that leads to death. Currently, the management of COVID-19 infections is mainly symptomatic, and new agents had been approved to treat COVID-19 infections including antiviral agents, such as remdesivir, baricitinib, and molnupiravir, and monoclonal antibodies such as tocilizumab and sotrovimab. However, these agents have many adverse effects, are relatively expensive, are not readily available, and require close monitoring (Cascella et al., 2022).

On the other hand, it is important to know that immune dysregulation and other factors such as immune dysfunction, old age, obesity, diabetes, stress, and depression have been shown to be the main leading cause of exacerbation of COVID-19 symptoms and increased mortality (Raslan et al., 2022a). Since the immune status plays an essential role in combating COVID-19, herbal medicines, which show immunomodulatory effects, may help prevent and even treat COVID-19.

The use of medicinal plants and their bioactive ingredients is considered a reasonable alternative therapy for a wide range of diseases, including cardiovascular diseases such as hypertension, coronary arterial disease, peripheral arterial, and cerebrovascular diseases (Al Disi et al., 2016; Anwar et al., 2016; Shaito et al., 2020); brain injury; and neurodegenerative disorders including Alzheimer’s disease, Parkinson’s disease, and amyotrophic lateral sclerosis (Safdari et al., 2021; Zahedipour et al., 2022); and aging (Phu et al., 2020). The therapeutic success of using herbal products for treating different diseases was mainly mediated through the enhancement of NO secretion by a rise in eNOS expression and activity and the reduction of reactive oxygen species (ROS) levels leading to normal vascular function, changing the way transcription factors (NF-κB) act, and suppressing pro-inflammatory mediators such as TNF-α (Anwar et al., 2016). Additionally, the potent therapeutic properties of herbal medicines and their ability to ameliorate pathological conditions associated with different diseases were demonstrated (Shaito et al., 2020); thus, their use in combination with conventional therapy may offer a solution in future preventative and curative strategies (Zahedipour et al., 2022).

Recently, there has been an interest toward the consumption of botanical medicines, which may have antiviral, anti-inflammatory, and immunomodulatory effects (Nugraha et al., 2020) as the use of natural products approved by the Egyptian Food and Drug Administration (Egyptian-FDA) might prove to have several therapeutic benefits superior to those of chemical compounds, including better safety, availability, affordability, and fewer requirements for monitoring.

During the COVID-19 pandemic, a number of countries incorporated complementary and alternative medicine (CAM) into their healthcare protocols, with a significant number of individuals using CAM interventions to improve immunity and prevent acute COVID-19 infection (Kim et al., 2022). Accordingly, molecular pathogenesis is considered an important factor for the selection of effective preventive measures and the development of targeted therapies against COVID-19 (Rostami et al., 2022). Consequently, some medicinal plants have shown promising effects against COVID-19 disease including resveratrol (Ahmadian et al., 2021), *Glycyrrhiza*, *Zingiber*, cassia, and *Nigella sativa* (Ramazani et al., 2021). In addition, they possess good ability to target proteins and certain stages of the virus life cycle in addition to its anti-thrombotic, anti-apoptotic, anti-inflammatory, and antioxidant properties in alveolar spaces (Giordo et al., 2021).

*Nigella sativa*, a widely used medicinal plant of the family Ranunculaceae and commonly known as black cumin/kalonji, has been traditionally consumed for the treatment of constitutional symptoms associated with respiratory viral infections, including fever, cough, bronchitis, and shortness of breath, in addition to its role in the management of a variety of acute and chronic conditions, including skin allergy, rheumatic pain and inflammation, hypertension, diabetes, liver diseases, mental disorder, anorexia, insomnia, and dysmenorrhea (Hannan et al., 2021). Many clinical studies have investigated its therapeutic potential and provided evidence that supports its antioxidant, analgesic, anti-inflammatory, antimicrobial, anthelmintic, antidiabetic, cardioprotective, neuroprotective, hepatoprotective, gastroprotective, nephroprotective, anti-oxytocic, anti-obesity, anticancer, anti-asthmatic, immunomodulatory, and wound-healing activities (Yimer et al., 2019).

The main active principles found in *Nigella sativa* are the volatile oil thymoquinone, saponin α-hederin, and alkaloid nigellidine. It is worth mentioning that thymoquinone had an anti-inflammatory effect through the suppression of prostaglandins; leukotrienes release; and upregulation of the levels of IL-4 and IL-10, the anti-inflammatory cytokines (Khazdair et al., 2021). In addition, it has an immunomodulation effect through activation of T cells, promotes the production of IFN-γ, and has a strong ability to inhibit the main protease (MPRO) in SARS-CoV-2 (Abdallah et al., 2022). Moreover, it was reported that *Nigella sativa* could prevent the release of histamine and effectively reduce inflammation on mast cells while having no deleterious effects on these cells. In addition, it was found, through a molecular docking study, that nigellidine and α-hederin inhibited the main proteases (3CLpro/Mpro) of SARS-CoV-2 (Bouchentouf and Missoum, 2020; Ullah et al., 2022)*.* Furthermore, α-hederin demonstrates anti-inflammatory and bronchodilator properties *via* increasing cAMP levels, which increases β2-adrenergic responsiveness. Therefore, *Nigella sativa* has positive implications due to its immunomodulatory, antioxidant, antiviral, and anti-inflammatory properties (Imran et al., 2022)*.* It was reported that the acute respiratory distress syndrome together with the cytokine storm is the leading cause of death in COVID-19 patients (Khazdair et al., 2021; Shirvani et al., 2021) and that more clinical data are required to investigate the efficacy and potency of *Nigella sativa* in COVID-19 (Khazdair et al., 2021; Shirvani et al., 2021). Based on the aforementioned beneficial mechanisms of actions of *Nigella sativa*, it could be considered an attractive candidate for an add-on therapy for the management of COVID-19 infection.

It is worth mentioning that diets containing cereals, fruits, vegetables, and whole-grain cereal are the main contributors to restoring gut microbiota balance; on the other hand, diets containing high fat content are unfavorable due to their negative effect on microbiota diversity. Thus, massive use of antibiotics is not recommended as it was proved to affect gut microbiota abundance and balance, leading to several inflammatory diseases; instead, probiotics and prebiotics have been proven to regulate the bacterial balance and reduce the probability of bacterial and viral infections (Raslan et al., 2022b). Additionally, the adequacy of vitamin D3 dietary intake and comparing it to a dietary reference intake is important; there is a need to increase awareness about the importance of vitamin D3 in children’s diet (Fahmy et al., 2016) specifically and in the whole community generally since it is a very important supplement, and its deficiency is responsible for the pathogenesis and increasing incidence probabilities of several diseases.

Studies conducted on different groups of participants have shown that those with lower levels of 25-hydroxyvitamin D3 are at higher risk of acute respiratory infection (Meltzer et al., 2020). In addition, low vitamin D3 levels have been associated with an increase in inflammatory cytokines and a significantly increased risk of pneumonia. It is well-known that the principal defense against uncontrolled inflammation and viral infection is provided by T regulatory lymphocytes (Tregs) whose levels were reported to be low in many COVID-19 patients; the levels can be increased by vitamin D3 supplementation (Kenneth Weir et al., 2020).

Additionally, vitamin D3 supplementation could improve the function of macrophages and dendritic cells, which may augment the innate and adaptive immune systems (Bilezikian et al., 2020). Martineau and Forouhi (2020) and Mitchell (2020) suggested that optimal serum levels of vitamin D3 may have immunomodulatory and anti-inflammatory effects, which may benefit patients with COVID-19. In addition, vitamin D3 supplementation might enhance the recovery of COVID-19 patients or decrease disease severity (Stroehlein et al., 2021).

The objective of this study was to investigate and compare the effect of *Nigella sativa*, vitamin D3, and their combination as add-on therapy in addition to the standard therapy versus standard therapy alone on the viral clearance and alleviation of COVID-19 symptoms.

## Patients and methods

### Study design

This was a randomized, controlled, open-label study that was conducted at the Respiratory Hospital at Kobry El Qobba Armed Forces Medical Complex after the approval of the local institutional review board.

### Ethics

The study protocol was approved by the Ethics Committee, Faculty of Pharmacy, Ain Shams University, registered at the Egyptian Ministry of Health (MOH). Written informed consent was obtained from each patient before enrollment in the study without any obligations to complete the study if they did not want to. All aspects of this study followed the ethical standards of the Declaration of Helsinki (World Medical Association, 2013). The study was registered on clinicaltrials.gov (NCT ID: NCT04981743).

### Study subjects and treatment

Patient recruitment was conducted according to the inclusion and exclusion criteria set in the study design before starting. Accordingly, 120 SARS2-CoV-2 polymerase chain reaction (PCR)-positive patients who fulfilled the inclusion criteria were included in the study. The inclusion criteria were as follows: age 18–65 years and patients with mild to moderate COVID-19 symptoms according to the classification of Gandhi et al. (2020).

Patients were excluded from the study for any of the following criteria: severe illness requiring admission to the intensive care unit; asymptomatic chronic kidney disease (i.e., estimated glomerular filtration rate [eGFR] < 30 ml/min); end-stage renal disease requiring dialysis; severe chronic liver disease (alanine transaminase [AlT] or aspartate transaminase [AST] > 5 times the upper limit of normal); and contraindications or allergy to any of the interventional drugs, pregnancy, and breastfeeding.

**FIGURE 1:**
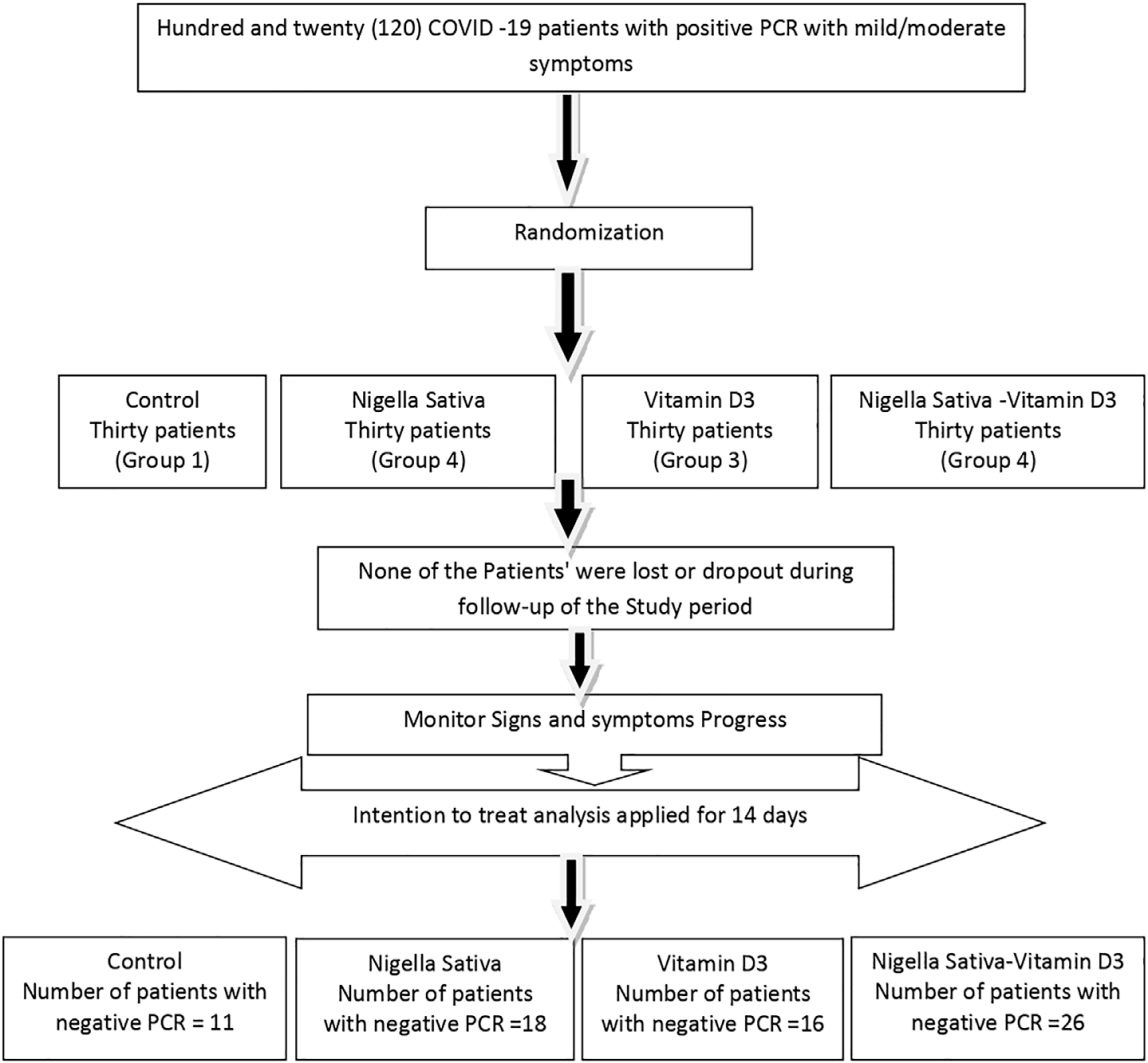
CONSORT diagram of the study.

Patients were randomly assigned into one of the following four groups, 30 patients in each: Group (1) patients received a total oral dose of 900 mg *Nigella sativa* (Baraka^®^, Pharco Pharmaceuticals, Cairo, Egypt) in the form of soft gelatin capsules containing 450 mg, given as two capsules twice daily for two weeks (Mahdavi et al., 2015; Ashraf et al., 2021) in addition to the standard therapy. Group (2) patients received 2,000 IU of vitamin D3 (Davalindi^®^, Medical Union Pharma, Cairo, Egypt) in the form of 1000-IU tablets given as two tablets, once daily, in addition to the standard therapy. Group (3) patients received 900 mg of *Nigella sativa* and 2000 IU of vitamin D3 in the same manner of dosing as in the previous groups in addition to the standard therapy. In Group 4), which is the control group, patients received the standard therapy of COVID-19 (Masoud et al., 2020), which was as follows: 500 mg azithromycin, one capsule once daily for 5 days; 1 g vitamin C, one capsule once daily; 50 mg zinc, one capsule once daily; 100-mg sachets of lactoferrin, twice daily; 10-mg rivaroxaban tablet, once daily in moderate cases as per their computed tomography (CT) findings; and 500-mg paracetamol tablets as antipyretic. The allocation of subjects to the four study arms and study design is shown in the CONSORT diagram (Figure 1).

### Outcome evaluation

The main outcomes and primary endpoint of this study were both the viral clearance indicated by a negative polymerase chain reaction (PCR) and the alleviation of symptoms during the study follow-up duration of 14 days.

Patients’ clinical symptoms were evaluated daily, and they underwent PCR on the 7th and 14th day of therapy. If any of the patients tested negative on day 7, they were considered to have been cleared of the virus, the 14th day PCR test was not performed, and further follow-up was deferred. However, if the patient tested gave a positive PCR result, follow-up was continued until the PCR test on day 14 was done, and if negative, patients were again considered to have been cleared of the virus. However, if still positive at day 14, patients continued on standard care therapy with neither further administration of the interventional therapy nor follow-up.

The clinical response to therapy was denoted by a significant reduction of fever on day 3, reduction of fatigue on day 4, relief from headache on day 5, absence of rhinorrhea and dyspnea on day 7, and improvement of cough from productive to dry/absent or from dry to absent on day 7. Additionally, laboratory findings, including C-reactive protein (CRP), oxygen saturation (PO_2_), hemoglobin levels, total leukocytic count, lymphocyte count, and serum ferritin, were recorded at baseline and on the 7th day of therapy. A computed tomography (CT) scan was done at baseline to assess the severity of pneumonia evidenced by ground-glass opacity.

### Statistical analysis

Statistical analysis was performed using R software version 4.1.1. Two-sided *p*-values of less than 0.05 were considered statistically significant. The normality assumption was tested by the Shapiro–Wilk test; parametric data were represented by means and standard deviations, and were compared using one-way ANOVA. Non-parametric data were represented by medians and ranges, and were compared using the Mann–Whitney U or Kruskal–Wallis tests. Multiple linear regression analyses were performed to quantify the marginal effect of treatments on vital signs and laboratory parameters while controlling for the pre-treatment values. Ordinal and binary logistic regressions were performed to study the effect of treatments on signs and symptoms while adjusting for baseline signs and symptoms. Outliers were not omitted from any of the regression models.

### Sample size calculation

The sample size was calculated by using GPower v.3.1.9.4 (Erdfelder et al., 1996). According to Connelly (2008), extant literature suggests that a pilot study’s sample size should be around 10% of the sample project for the larger parent study. Expecting a small effect size of 0.10, to achieve a power of 80% to reject the null hypothesis, with four equally sized groups and a type 1 error rate (alpha) of 0.05, the required sample size was calculated to be 110 participants to detect and quantify the aforementioned effect. To account for dropouts, we decided to recruit 120 participants, equally divided into four groups (thirty participants in each group).

## Results

A total of 120 patients were enrolled in the current study, where the median age of participants was 41.5 years (range: 19–64 years). A total of 70 patients were males (58.3%), of which four patients were diabetic, 10 patients were hypertensive, and one patient was a smoker. Patient demographics and baseline characteristics are shown in Table 1.

**TABLE 1:** Patients’ demographics and baseline characteristics among the studied groups.

	STD (N = 30) n (%)	NS (N = 30) n (%)	VD (N = 30) n (%)	NS + VD (N = 30) n (%)	*p*-value
Age (years):					0.16^1^
Median (range): 41.5 (19–64)	26.0 (21.0–64.0)	29.0 (21.0–62.0)	50.0 (20.0–64.0)	44.5 (19.0–63.0)	
Gender:					0.33^2^
Female (50, 41.7%)	9 (30.0)	12 (40.0)	13 (43.3)	16 (53.3)	
Male (70, 58.3%)	21 (70.0)	18 (60.0)	17 (56.7)	14 (46.7)	
DM history:					0.19^3^
Absent (116)	30 (100.0)	27 (90.0)	29 (96.7)	30 (100.0)	
Present (4)	0 (0.0)	3 (10.0)	1 (3.3)	0 (0.0)	
HTN:					0.22^3^
Absent (110)	30 (100.0)	26 (86.7)	27 (90.0)	27 (90.0)	
Present (10)	0 (0.0)	4 (13.3)	3 (10.0)	3 (10.0)	
Smoking history:					1.00^3^
Non-smoker (119)	30 (100.0)	30 (100.0)	29 (96.7)	30 (100.0)	
Smoker (1)	0 (0.0)	0 (0.0)	1 (3.3)	0 (0.0)	

^1^ Kruskal–Wallis rank-sum test.

^2^ Pearson’s chi-squared test.

^3^ Fisher’s exact test for count data.

Abbreviations: DM, diabetes mellitus; HTN, hypertension; NS, *Nigella sativa*; STD, standard therapy; VD, vitamin D3.

Patients in all treatment arms were matched for age, sex, comorbidities, and smoking history at baseline. All arms were matched for baseline signs and symptoms except for headache: patients randomized to receive standard therapy alone (controls) had a significantly higher prevalence of headache (Table 2). Regarding ferritin and lymphocytes, patients randomized to either *Nigella sativa* alone or the *Nigella sativa*–vitamin D3 combination showed a significantly higher ferritin level and lower lymphocyte count at baseline (Table 3).

**TABLE 2:** Baseline signs and symptoms of the patients among the studied groups.

	STD (N = 30)	NS (N = 30)	VD (N = 30)	NS + VD (N = 30)	*p*-value
Cough, n (%)					0.93^2^
Absent	6 (20.0)	5 (16.7)	9 (30.0)	8 (26.7)	
Dry	19 (63.3)	20 (66.7)	17 (56.7)	17 (56.7)	
Productive	5 (16.7)	5 (16.7)	4 (13.3)	5 (16.7)	
Diarrhea, n (%)	11 (36.7)	9 (30.0)	4 (13.3)	7 (23.3)	0.20^1^
Fatigue, n (%)	30 (100.0)	28 (93.3)	29 (96.7)	27 (90.0)	0.52^2^
Headache, n (%)	23 (76.7)^*^	16 (53.3)	13 (43.3)	14 (46.7)	0.04^2^
Rash, n (%)	1 (3.3)	0 (0.0)	0 (0.0)	2 (6.7)	0.62^2^
Conjunctivitis, n (%)	2 (6.7)	5 (16.7)	1 (3.3)	2 (6.7)	0.39^2^
Rhinorrhea, n (%)	7 (23.3)	12 (40.0)	11 (36.7)	10 (33.3)	0.55^1^
Anosmia, n (%)	14 (46.7)	17 (56.7)	14 (46.7)	14 (46.7)	0.83^1^
SOB, n (%)	10 (33.3)	12 (40.0)	9 (30.0)	16 (53.3)	0.26^1^
Pharyngitis, n (%)	17 (56.7)	12 (40.0)	9 (30.0)	15 (50.0)	0.17^1^
Ageusia, n (%)	15 (50.0)	15 (50.0)	14 (46.7)	15 (50.0)	0.99^1^
Vomiting, n (%)	3 (10.0)	4 (13.3)	3 (10.0)	3 (10.0)	1.00^2^

^1^ Pearson’s chi-squared test.

^2^ Fisher’s exact test.

Abbreviations: NS, *Nigella sativa*; SOB, shortness of breath; STD, standard therapy; VD, vitamin D3.

**TABLE 3:** Baseline values of the laboratory parameters for the studied groups.

Parameter	STD (N = 30)	NS (N = 30)	VD (N = 30)	NS + VD (N = 30)	*p*-value*
CRP, ng/dl					0.55
Median (range):	19.5 (1.00–119)	15.0 (0.50–150)	23.0 (2.0–70.0)	20.5 (2.0–200)	
Ferritin, ng/ml					<0.01**
Median (range):	185.5 (15.0–531)	250* (10.0–850)	195.0 (17.0–376.0)	318* (64.0–1000)	
Hemoglobin, g/dl					0.67
Median (range):	12.8 (8.50–15.3)	11.9 (9.00–16.0)	12.6 (9.3–15.6)	11.6 (9.00–14.8)	
TLC, × 10^3^/µl					0.28
Median (range):	3.80 (1.90–12.0)	3.30 (1.70–10.6)	4.0 (1.9–6.5)	3.40 (1.70–9.80)	
Lymph, /µl					<0.01**
Median (range):	1118 (700–2666)	1090* (550–2500)	1265.5 (700.0–1752.0)	895* (540–1800)	
SO_2_ (%)					0.84
Median (range):	97.0 (88.0–99.0)	98.0 (83.0–100)	97.0 (92.0–99.0)	97.0 (80.0–99.0)	
Temperature, °C					0.29
Median (range):	38.0 (37.0–40.0)	38.0 (37.0–39.5)	38.0 (37.0–40.0)	38.0 (37.0–40.0)	

*Kruskal–Wallis rank-sum test.

** Significant difference

Abbreviations: CRP, C-reactive protein; Lymph, lymphocytes; NS, *Nigella sativa*; SOB, shortness of breath; STD, standard therapy; SO_2_, oxygen saturation; TLC, total leukocyte count; VD, vitamin D3; ng, nanogram; µl, microliter; ml, milliliter; dl, deciliter.

Bold value means Significant difference.

### Improvement of signs and symptoms

Regarding the improvement of the signs and symptoms at the end of the study among the studied groups, the data showed that groups 1, 2, and 3 had reduced severity of cough, diarrhea, fatigue, and pharyngitis compared to Group 4 (the control group). However, the four treatment arms showed non-significant improvement in headache, rhinorrhea, anosmia, shortness of breath (SOB), ageusia, and vomiting, while there was a significant virological response on days 7 and 14 post-treatment (Table 4).

**TABLE 4:** Signs, symptoms, and values of polymerase chain reaction tests at the end of the study among the studied groups.

	STD (N = 30) n (%)	NS (N = 30) n (%)	VD (N = 30) n (%)	NS + VD (N = 30) n (%)	*p*-value
Cough (Day +7)					**0.02** ^2^
Absent	8 (26.7)	11 (36.7)	10 (33.3)	15 (50.0)	
Dry	11 (36.7)	18 (60.0)	16 (53.3)	11 (36.7)	
Productive	11 (36.7)	1 (3.3)	4 (13.3)	4 (13.3)	
Diarrhea	8 (26.7)	2* (6.7)	1* (3.3)	1* (3.3)	**0.01** ^2^
Fatigue (Day +4)	25 (83.3)	12* (40.0)	20* (66.7)	9* (30.0)	**<0.01** ^1^ **
Headache	14 (46.7)	9 (30.0)	14 (46.7)	6 (20.0)	0.08^1^
Rash	0 (0.0)	0 (0.0)	0 (0.0)	0 (0.0)	-
Conjunctivitis	0 (0.0)	1 (3.3)	0 (0.0)	0 (0.0)	1.00^2^
Rhinorrhea	4 (13.3)	6 (20.0)	9 (30.0)	3 (10.0)	0.24^2^
Anosmia	14 (46.7)	10 (33.3)	13 (43.3)	11 (36.7)	0.71^1^
SOB	8 (26.7)	5 (16.7)	6 (20.0)	4 (13.3)	0.60^2^
Pharyngitis	9 (30.0)	2* (6.7)	3* (10.0)	2* (6.7)	**0.02** ^1^
Ageusia	15 (50.0)	12 (40.0)	15 (50.0)	12 (40.0)	0.75^1^
Vomiting	1 (3.3)	0 (0.0)	0 (0.0)	0 (0.0)	1.00^2^
PCR positive (day 7)	26 (86.7)	15 (50.0)	25 (83.3)	14 (46.7)	**<0.01** ^2^ **
PCR positive (day 14)	19* (63.3)	12* (40.0)	14* (46.7)	4* (13.3)	**<0.01** ^1^ **

^1^ Pearson’s chi-squared test.

^2^ Fisher’s exact test.

*Significant difference.

Abbreviations: NS, *Nigella sativa*; PCR, polymerase chain reaction; SOB, shortness of breath; SO_2_, oxygen saturation; SOB, shortness of breath; STD, standard therapy; TLC, total leukocyte count; VD, vitamin D3.

Bold value means Significant difference.

**FIGURE 2:**
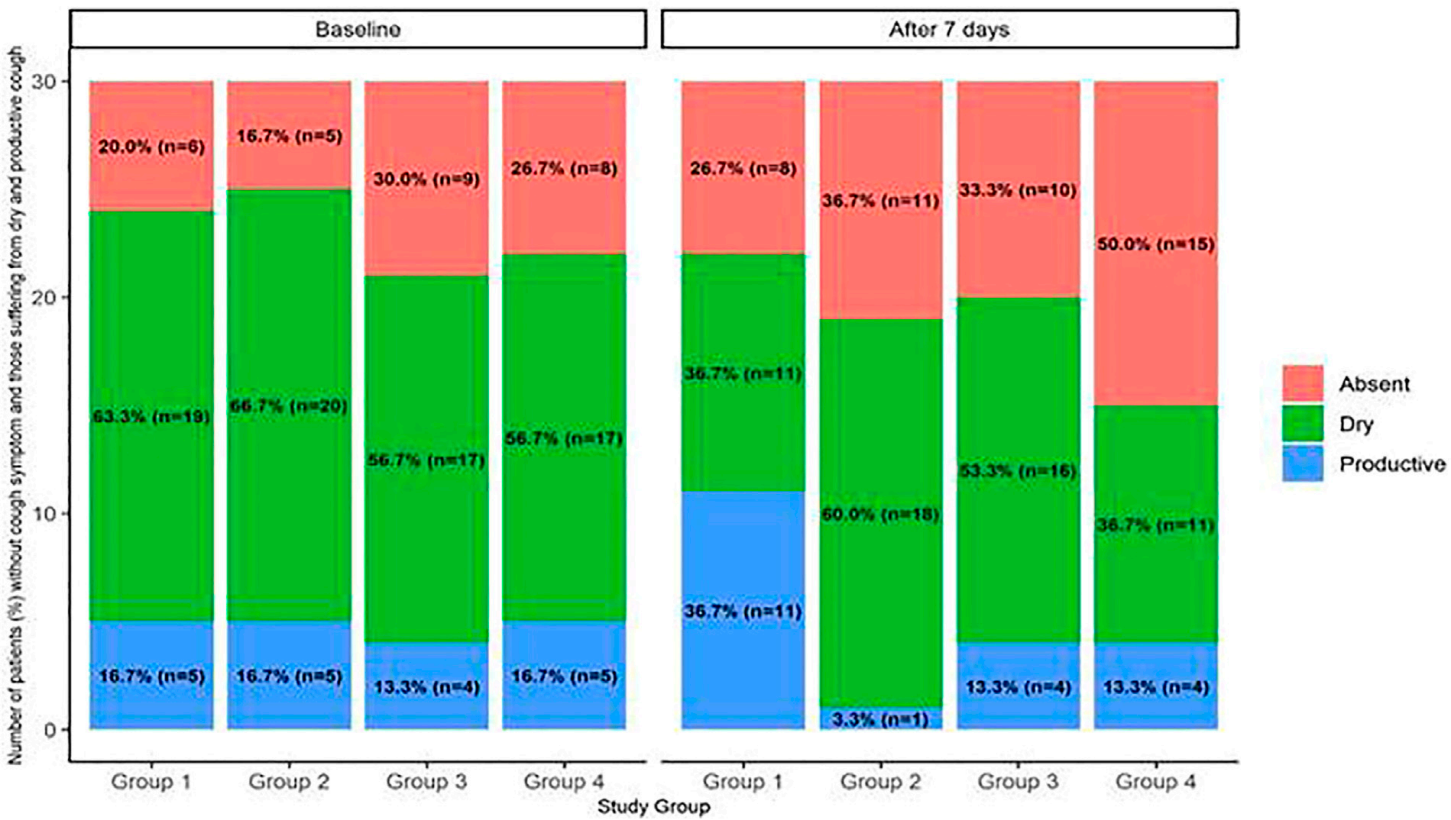
Stacked bar plot presenting the number (percentage) of patients corresponding to levels of cough severity among the studied groups, pre- and post-intervention. The bar plot presents the effect of the different study interventions on the levels of cough severity in patients of the four groups: (a) Group 1 received standard therapy (controls), Group 2 received NS, Group 3 received VD, and Group 4 received NS-VD combination. (b) Different levels of cough severity were defined in ascending order: absent, dry, and productive with the change in the percentage of patients (number in each group) detected pre- and 7 days post-intervention. (b) The figure clarified the remarkable decrement in the proportion of patients with productive cough among the studied groups, which revealed effect of the intervention. (c) Multiple logistic regression analysis used for evaluating the effect of the interventional therapy versus the standard therapy on cough severity showed that *Nigella sativa* independently reduced the odds of worsening of cough (odds ratio [OR] = 0.2, 95% CI = 0.07–0.59).

It was clear from the results shown in Table 5. Reference source not found that *Nigella sativa* independently reduced the odds of worsening cough [odds ratio (OR) = 0.2, 95% CI = 0.07–0.59], odds of post-treatment fatigue [odds ratio (OR) = 0.15, 95% CI = 0.04–0.48], and anosmia [odds ratio (OR) = 0.15, 95% CI = 0.02–0.74]. On the other hand, vitamin D3 had no significant impact on any symptom post-treatment. However, the *Nigella sativa*–vitamin D3 combination showed a reduction in the severity of cough [odds ratio (OR) = 0.23, 95% CI = 0.07–0.70] similar to that of the *Nigella sativa* group [odds ratio (OR) = 0.2, 95% CI = 0.07–0.59]. Moreover, Figure 2 represents the effect of the different interventions on the cough severity, reflected by the change in the number of patients suffering from severe cough pre- and post-treatment among the four studied groups. However, the combination significantly reduced diarrhea [odds ratio (OR) = 0.10, 95% CI = 0.01–0.66], fatigue [odds ratio (OR) = 0.10, 95% CI = 0.03–0.33], shortness of breath [odds ratio (OR) = 0.11, 95% CI = 0.02–0.58], and pharyngitis [odds ratio (OR) = 0.14, 95% CI = 0.02–0.70].

**TABLE 5:** Results of the multiple logistic regression analysis for evaluating the effect of interventional therapy versus standard therapy on patients’ symptoms.

Variable	Cough**	Diarrhea	Fatigue	Headache	Rhinorrhea	Ageusia	Anosmia	SOB	Pharyngitis
Interventional treatment (versus STD)									
NS (OR)	0.20**	0.20	0.15**	0.73	0.89	0.42	0.15*	0.23	0.18
CI	(0.07–0.59)	(0.03–0.92)	(0.04–0.48)	(0.22–2.40)	(0.16–5.07)	(0.08–1.86)	(0.02–0.74)	(0.04–1.23)	(0.02–0.94)
VD (OR)	0.48	0.13	0.44	2.75	2.62	1.25	0.67	0.58	0.44
CI	(0.17–1.34)	(0.01–0.82)	(0.12–1.50)	(0.79–10.8)	(0.48–16.2)	(0.27–6.01)	(0.11–3.90)	(0.09–3.47)	(0.07–2.29)
NS + VD (OR)	0.23*	0.10*	0.10***	0.44	0.39	0.42	0.33	0.11*	0.14*
CI	(0.07–0.70)	(0.01–0.66)	(0.03–0.33)	(0.12–1.53)	(0.05–2.52)	(0.08–1.86)	(0.05–1.79)	(0.02–0.58)	(0.02–0.70)

*p < 0.05 **p < 0.01 ***p < 0.001.

**Ordinal logistic regression model. OR, odds ratio; CI, confidence interval.

Abbreviations: NS, *Nigella sativa*, VD, vitamin D3; SOB, shortness of breath; STD, standard therapy; NS + VD, *Nigella sativa*–vitamin D3 combination.

### Measurements of laboratory data

Considering the results presented in Table 6, it was evident that the obtained post-treatment levels of C-reactive protein (CRP), ferritin, and hemoglobin values were comparable among the four studied groups. On the other hand, both Group 2 (*Nigella sativa* group) and Group 3 (vitamin D3 group) showed an increase in lymphocyte counts at the end of the study period. Moreover, the impact of treatments on the patient’s vital signs showed a comparable reduction in body temperature approaching normal level among the four arms of the study. Interestingly, Group 4, who received *Nigella sativa* were evaluated daily–vitamin D3 combination, showed a significant improvement in post-treatment oxygen saturation compared to the other studied groups.

**TABLE 6:** Values of laboratory parameters and vital signs at the end of the study among the studied groups.

Parameter	STD (N = 30)	NS (N = 30)	VD (N = 30)	NS + VD (N = 30)	*p*-value*
CRP, ng/dl					0.28
Median (range):	19.5 (6.0–99.0)	12.0 (2.0–95.0)	14.0 (2.0–115.0)	13.0 (0.5–170.0)	
Ferritin, ng/ml					0.07
Median (range):	194.0 (17.0–724.0)	210.0 (15.0–950.0)	219.0 (27.0–459.0)	290.0 (73.0–800.0)	
Hemoglobin, g/dl					0.58
Median (range):	12.7 (9.1–15.3)	13.0 (9.5–15.2)	12.6 (9.7–15.5)	11.5 (9.5–15.0)	
TLC, × 10^3^/µl					0.09
Median (range):	3.9 (2.5–12.7)	4.4 (2.9–11.0)	4.5 (2.3–15.0)	4.3 (2.3–7.9)	
Lymph, /µl					0.03
Median (range):	1046.0 (678.0–3086.0)	**1275.0** (700.0–3100.0)	**1332.5** (850.0–1931.0)	1130.0 (620.0–2200.0)	
SO_2_ (%)					0.02
Median (range):	97.0 (90.0–99.0)	97.0 (78.0–99.0)	96.0 (85.0–99.0)	97.0 (94.0–99.0)	
Temperature, °C (D3)					<0.01**
Median (range):	**37.5** (37.0–39.0)	**37.0** (37.0–38.5)	**37.0** (37.0–39.0)	**37.0** (37.0–38.0)	

*Kruskal–Wallis rank-sum test.

** Significance.

Abbreviations: CRP, C-reactive protein; Lymph, lymphocytes; NS, *Nigella sativa*; SO_2_, oxygen saturation; STD, standard therapy; TLC, total leukocyte count; VD, vitamin D3; NS + VD, *Nigella sativa*–vitamin D3 combination.

Bold value means Significant difference.

**FIGURE 3:**
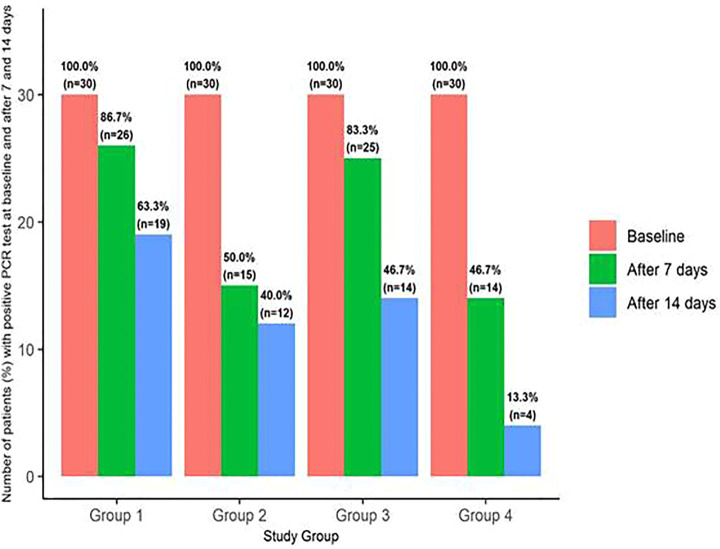
Bar plot presenting the percentage (number) of patients with positive polymerase chain reaction test at baseline and after 7 and 14 days among the studied groups. The bar plot indicated treatment failure, evidenced by absence of the viral clearance, detected by positive polymerase chain reaction (PCR) tests among the studied groups: (a) Group 1 received standard therapy (controls), Group 2 received NS, Group 3 received VD, and Group 4 received NS-VD combination. (b) Percentage (number) of patients with positive PCR test (no viral clearance) is presented on the bar chart corresponding to each study group pre- and at 7 and 14 days’ post-intervention. (c) Binary logistic regression analysis used for evaluating the effect of the interventional therapy versus the standard therapy on viral clearance evidenced by results of PCR showed that *Nigella sativa* alone and the *Nigella sativa*–vitamin D3 combination significantly reduced the odds of positive PCR at day 7 with odd ratios (OR) = 0.15 and 0.13 and 95% confidence interval (95% CI) = 0.04–0.51 and 0.03–0.45, respectively. However, only the combination significantly reduced the odds of a positive PCR at day 14 post-treatment (odds ratio [OR] = 0.09, 95% CI = 0.02–0.30).

The data presented in Table 7 showed that both *Nigella sativa* alone and the *Nigella sativa*–vitamin D3 combination significantly reduced the odds of positive PCR at day 7 with odds ratios (OR) = 0.15 and 0.13, with 95% confidence interval (95% CI) = 0.04–0.51 and 0.03–0.45, respectively. However, only the combination significantly reduced the odds of a positive PCR at day 14 post-treatment [odds ratio (OR) = 0.09, 95% CI = 0.02–0.30], and this is evident in Figure 3.

**TABLE 7:** Results of the logistic regression analysis of the results of polymerase chain reaction on day 7 and 14 among the studied groups.

	Follow-up PCR	
	Day 7	Day 14
Treatment (vs. STD):		
NS (OR)	0.15**	0.39
CI	(0.04–0.51)	(0.13–1.08)
VD (OR)	0.77	0.51
CI	(0.17–3.23)	(0.18–1.41)
NS + VD (OR)	0.13**	0.09***
CI	(0.03–0.45)	(0.02–0.30)

*p < 0.05 **p < 0.01 ***p < 0.001.

Abbreviations: NS, *Nigella sativa*; VD, vitamin D3; PCR, polymerase chain reaction; STD, standard therapy; NS + VD, *Nigella sativa*–vitamin D3 combination; OR, odds ratio; CI, confidence interval.

### Measurement of body temperature, oxygen saturation, total leukocyte count, ferritin, and lymphocytes

It is clear from the results presented in Table 8 that Group 2 (*Nigella sativa*) showed an increase in values of lymphocytes by an average of 327 × 10^3^/µL and a decrease in the temperature by 0.53°C compared to Group 1 (controls). In addition, it was apparent that Group 3 (vitamin D3) showed an increase in the total leukocyte count (TLC) by 1.17 × 10^3^/µL and lymphocytes by 137/µL, and the temperature reduced by only 0.44°C at the end of the study. Conversely, vitamin D3 (Group 3) was correlated with a modest decrease in oxygen saturation by 1.48%. Finally, Group 4 (*Nigella sativa* and vitamin D3 combination) showed a decrease in ferritin levels by 49.2 ng/ml, an increase in lymphocytes by an average of 249/µL, and a decrease in body temperature by 0.56°C.

**TABLE 8:** Multiple linear regression analysis for evaluating the impact of treatments on post-treatment laboratory parameters versus standard treatment while adjusting for baseline values.

Variable	Lab parameter, estimate (95% CI)						
	CRP (ng/dl)	Ferritin (ng/ml)	Hb (g/dl)	TLC (×10^3^/µl)	Lymph (/µl)	SO_2_ (%)	T (°C)
Treatment (vs. STD):							
NS (OR)	−6.57	−33.07	0.13	0.63	327***	−0.53	−0.53***
CI	(−13.17–0.02)	(−70.1–3.95)	(−0.26–0.52)	(−0.08–1.34)	(192–462)	(−1.45–0.39)	(−0.75–0.32)
VD (OR)	1.02	−4.24	0.05	1.17**	137*	−1.48**	−0.44***
CI	(−5.57–7.61)	(−41.0–32.5)	(−0.35–0.44)	(0.47–1.88)	(2.30–272)	(−2.40–0.56)	(−0.65–0.23)
NS + VD (OR)	−4.48	−49.3**	−0.09	0.57	249***	0.53	−0.56***
CI	(−11.1–2.18)	(−88.2–10.3)	(−0.48–0.30)	(−0.13–1.28)	(107–390)	(−0.39–1.44)	(−0.77–0.35)
Observation:	120	119	120	120	120	120	120
R^2^	0.81	0.83	0.81	0.63	0.67	0.58	0.37
Adjusted R^2^	0.80	0.82	0.80	0.61	0.66	0.56	0.35

*p < 0.05, **p < 0.01, ***p < 0.001.

Abbreviations: CRP, C-reactive protein; Hb, hemoglobin; Lymph, lymphocytes; NS, *Nigella sativa*; SO_2_, oxygen saturation; STD, standard therapy; TLC, total leukocyte count; T, temperature; VD, vitamin D3; NS + VD, *Nigella sativa*–vitamin D3 combination; OR, odds ratio; CI, confidence interval; R^2^, regression coefficient.

## Discussion

Despite the development of different vaccines from Moderna and Pfizer-BioNTech and new drugs such as remdesivir and its combination with repurposed drugs such as chloroquine, hydroxychloroquine (Younis et al., 2020), and ivermectin (Wehbe et al., 2021; Niranjan et al., 2022), which act on different enzymes and proteins of SARS-CoV-2 including helicase, main protease, non-structural proteins (Nsp15 and Nsp9), RNA-dependent-RNA-polymerase, and RNA replicate, the fight against COVID-19 has not yet ended (Alshrari et al., 2022; Chatterjee and Thakur 2022). Additionally, corticosteroids caused suppression of the excessive immunological response with the prevention of multi-organ failure and death in severe cases (Sen et al., 2022). Moreover, ACEIs/ARBs, statins, SGLT-2 inhibitors, and aldosterone receptor antagonists may play a crucial role in mitigating cardiovascular risk and morbidity in COVID-19 patients (Wehbe et al., 2020). However, the development of new SARS-CoV-2 strains, such as α, β, γ, δ, and Omicron, creates further problems for existing vaccines and the treatment of COVID-19 (Asdaq et al., 2021). Therefore, the use of complementary and alternative medicines (CAM) including *Nigella sativa* and vitamin D3 might be a promising strategy in the management of COVID-19.

Globally, there is increasing use of herbal medicines and natural supplements due to the belief that they are always safe and carry no risk because they are from natural sources (Awodele et al., 2018). Herbal prescriptions are used for the treatment of various diseases, and the vast majority of people use these products as first-line remedies. Thus, attention has been drawn to claims by practitioners on the effectiveness of herbal medicines for the prevention and cure of COVID-19 (Awodele et al., 2018).

In the current study, it was found that the administration of *Nigella sativa*, with or without vitamin D3, as add-on therapy in the management of COVID-19 was superior to the standard therapy alone or the standard therapy with vitamin D3 in terms of early viral clearance and alleviation of signs, symptoms, and the laboratory parameters relevant to COVID-19. It is worth mentioning that the present study is the first of its kind in terms of study design, investigational agents, and comparator groups.

The findings of the present study suggested the superiority of the *Nigella sativa*–vitamin D3 combination group (Group 4) compared to other groups, evidenced by the significant alleviation of COVID-19 signs and symptoms as follows: 50% of patients were free of cough after 7 days, 70% of patients did not have fatigue after 4 days, 80% of patients were relieved of headache after 5 days, 90% of patients showed no rhinorrhea after 7 days, 86.7% of patients had no dyspnea after 7 days, the median temperature reduced after 3 days of treatment, and the proportion of negative results of the polymerase chain reaction test on the 7th and 14th day of therapy compared to the other three studied groups was high. These results suggested the superiority of the combination, supported by faster viral clearance through the inhibition of TANK-binding kinase 1 (TBK1), together with the downregulation of the interferon regulatory factor 3 (IRF-3) activation, which has a critical role in viral and bacterial innate immune responses through regulation of type I interferon production (Aziz et al., 2018).

In addition, using *Nigella sativa* as add-on therapy in COVID-19 might be attributed to cyclooxygenase and lipooxygenase pathways inhibition, which in turn contribute to worsening of respiratory distress.

In a clinical study conducted on patients infected with COVID-19, a better clinical outcome and earlier viral clearance were reported in those who received a combination of *Nigella sativa* and honey attacking multiple sites of SARS-CoV-2 (Ashraf et al., 2020). In addition, it was reported that there was a prophylactic potential of *Nigella sativa* when combined with honey against COVID-19 (Ashraf et al., 2021). In addition, Koshak et al. (2021) reported that *Nigella sativa* supplementation was correlated with faster symptom alleviation than the standard therapy in patients with mild COVID-19.

The role of *Nigella sativa* as add-on therapy in COVID-19 was strengthened by its anti-allergic, antioxidant, and anti-asthmatic effects in addition to its documented immunomodulatory activity, where it activates natural killer cells, interferon release, and dendritic cells and suppresses virus multiplication by its chemical constituents (Haddadi and Pour, 2022). Thymoquinone, which is the major constituent of *Nigella sativa*, demonstrated a promising anti-inflammatory, cytotoxic, antimicrobial, antiviral, antioxidant, immunostimulant, and anticoagulant effects (Abdallah et al., 2022); its anti-inflammatory effect is mediated through the suppression of prostaglandins and leukotriene release including COX-2, TNF-α, IL-6, and upregulation of the levels of IL-4 and IL-10, the anti-inflammatory cytokines (Khazdair et al., 2021).

It is well-known that *Nigella sativa* is a source of zinc (Muhammad, 2021), which has shown activity against SARS-CoV-2 (Jalal et al., 2021), indicating that *Nigella sativa* and its various compositions have all the attributes to be used as a promising add-on therapy for the management of COVID-19 among high-risk individuals as well as to treat mild cases of COVID-19.

The current study showed that the effect of vitamin D3 supplementation seems to be additive when combined with *Nigella sativa* and modifies the course of the disease for viral clearance without improvement of patients’ symptoms; these findings were in agreement with those of Rastogi et al. who reported that a greater proportion of vitamin D3-deficient individuals with SARS-CoV-2 infection turned SARS-CoV-2 negative with a significant decrease in fibrinogen on a high dose of vitamin D3 supplementation (Rastogi et al., 2022).

The increased production of Th1 pro-inflammatory cytokines resulting from the cytokine storm is one of the mechanisms through which SARS-CoV-2 damages lung tissue, leading to the development of acute respiratory failure (Lai et al., 2020). It is worth mentioning that vitamin D3 modulates both innate and adaptive immunity and reduces the cytokine storm by reducing concentrations of pro-inflammatory cytokines: IL-6, IL-8, IL-12, interferon-gamma (IFN-g), tumor necrosis factor alpha (TNFa), and IL-17 and by increasing the concentrations of the anti-inflammatory cytokines, IL-10, and Th2 cytokines, IL-4 and IL-5 (Gorman et al., 2012; Greiller and Martineau, 2015). Moreover, it was reported that vitamin D3 affects Th cell polarization by inhibiting Th1 (IFN-gamma release) and promoting Th2 cell development with production of their specific anti-inflammatory cytokines (Boonstra et al., 2001).

Concerning the role of vitamin D3, it was reported that SARS-CoV-2 infections lead to downregulation of ACE2 causing toxic accumulation of angiotensin II, which in turn contributed to acute respiratory failure. On the other hand, vitamin D3 was found to attenuate the effects of these interactions between SARS-CoV-2 and the renin–angiotensin–aldosterone system (RAAS) (Giménez et al., 2020), where it acts as a negative endocrine regulator which protects against acute lung injury and acute respiratory failure (Malek Mahdavi, 2020).

It is worth mentioning that the findings of the current study were in accordance with those presented by Murai et al. (2021), who reported that a single high dose of vitamin D3 showed a minimal decrease in length of hospital stay. Moreover, according to the investigation results of the study performed by Leaf and Ginde (2021), it was recommended not to use high doses of vitamin D3 in order to obtain effective clinical outcomes (Leaf and Ginde, 2021).

## Limitations

The current study has certain limitations, which are as follows: the evaluation of the effect of vitamin D3 through measurement of serum level of [25(OH)D] levels was not performed and was totally based on the clinical outcome of the patients. Additionally, the polymerase chain reaction test (PCR) was conducted at baseline and after 7 days of treatment only, which was not repeated on day 14 of the study except in the positive cases only in the current study.

## Conclusion

The current work confirms the clinical benefits of *Nigella sativa* and vitamin D3, and to the best of our knowledge, no study has evaluated the effect of this combination up to date. The therapeutic benefits of administration of the *Nigella sativa*–vitamin D3 combination as an add-on therapy were superior to using each of them separately, which was evidenced by the remarkable viral clearance in a shorter time interval accompanied by a reduction in the severity and progression of patients’ signs and symptoms. Thus, it is recommended to use the *Nigella sativa*–vitamin D3 combination as add-on therapy in the management of hospitalized COVID-19 patients and in-home isolated patients.

## Data Availability

The original contributions presented in the study are included in the article/Supplementary Material; further inquiries can be directed to the corresponding author.

## References

[B1] Abdallah H. M. El-Halawany A. M. Darwish K. M. Algandaby M. M. Mohamed G. A. Ibrahim S. R. M. (2022). Bio-guided isolation of SARS-CoV-2 main protease inhibitors from medicinal plants: *In vitro* assay and molecular dynamics. Plants 11 (15), 1914. 10.3390/PLANTS11151914 35893619 PMC9332707

[B2] Ahmadian R. Biganeh H. Panahi Y. Guest P. C. Jamialahmadi T. Sahebkar A. (2021). Resveratrol as a probable multiheaded treatment approach for COVID-19. Adv. Exp. Med. Biol. 1328, 441–446. 10.1007/978-3-030-73234-9_29 34981495

[B3] Al Disi S. S. Anwar M. A. Eid A. H. (2016). Anti-hypertensive herbs and their mechanisms of action: Part I. Front. Pharmacol. 19 (6), 323. 10.3389/fphar.2015.00323 PMC4717468 26834637

[B4] Alshrari A. S. Hudu S. A. Imran M. Asdaq S. M. B. Ali A. M. Rabbani S. I. (2022). Innovations and development of COVID-19 vaccines: A patent review. J. Infect. Public Health 15 (1), 123–131. 10.1016/j.jiph.2021.10.021 34742639 PMC8539827

[B5] Anwar M. A. Al Disi S. S. Eid A. H. (2016). Anti-hypertensive herbs and their mechanisms of action: Part II. Front. Pharmacol. 7, 50. 10.3389/fphar.2016.00050 27014064 PMC4782109

[B6] Asdaq S. M. B. Rajan A. Damodaran A. Kamath S. R. Nair K. S. Zachariah S. M. (2021). Identifying mucormycosis severity in Indian COVID-19 patients: A nano-based diagnosis and the necessity for critical therapeutic intervention. Antibiotics 10 (11), 1308. 10.3390/antibiotics10111308 34827246 PMC8615244

[B7] Ashraf S. Ashraf S. Akmal R. Ashraf M. Kalsoom L. Maqsood A. (2021). Prophylactic potential of honey and nigella sativa L. Against hospital and community-based SARS-CoV-2 spread: A structured summary of a study protocol for a randomised controlled trial. Trials 22 (1), 618. 10.1186/s13063-021-05510-3 34526081 PMC8441040

[B8] Ashraf S. Ashraf S. Ashraf M. Imran M. A. Kalsoom L. Siddiqui U. N. Farooq I. (2020). “Efficacy of honey and nigella sativa against COVID-19: HNS-COVID-PK trial,” in A multi-center placebo-controlled randomized clinical trial. medRxiv. 10.1101/2020.10.30.20217364 36420866

[B9] Awodele O. Oreagba I. Fajemirokun O. Samuel T. Coulidiaty G. Popoola T. D. (2018). Herbal medicines use : Remedies or risks. West Afr. J. Pharm. 29 (2), 1–18.

[B10] Aziz N. Son Y. J. Cho J. Y. (2018). Thymoquinone suppresses IRF-3-mediated expression of type I interferons via suppression of TBK1. Int. J. Mol. Sci. 19 (5), 1355. 10.3390/IJMS19051355 29751576 PMC5983753

[B11] Bilezikian J. P. Bikle D. Hewison M. Lazaretti-Castro M. Formenti A. M. Gupta A. (2020). Mechanisms in endocrinology: Vitamin D and COVID-19. Eur. J. Endocrinol. 183 (5), R133–R147. 10.1530/EJE-20-0665 32755992 PMC9494342

[B12] Boonstra A. Barrat F. J. Crain C. Heath V. L. SavelkoulO'Garra H. F. A. (2001). 1alpha, 25-Dihydroxyvitamin d3 has a direct effect on naive CD4(+) T cells to enhance the development of Th2 cells. J. Immunol. 167, 4974–4980. 10.4049/jimmunol.167.9.4974 11673504

[B13] Bouchentouf S. Missoum N. (2020). Identification of compounds from *Nigella sativa* as new potential inhibitors of 2019 novel coronasvirus (Covid-19): Molecular docking study. ChemRxiv. Cambridge: Cambridge Open Engage. 10.20944/PREPRINTS202004.0079.V1

[B14] Cascella M. Rajnik M. Aleem A. Dulebohn S. C. Di Napoli R. (2022). Features, evaluation, and treatment of coronavirus (COVID-19). *StatPearls*. Available at: https://www.ncbi.nlm.nih.gov/books/NBK554776/ (Accessed: February 21, 2022). 32150360

[B15] Chatterjee B. Thakur S. S. (2022). Remdesivir and its combination with repurposed drugs as COVID-19 therapeutics. Front. Immunol. 13, 830990. 10.3389/fimmu.2022.830990 35634324 PMC9134007

[B16] Connelly L. M. (2008). Pilot studies. Medsurg Nurs. 17 (6), 411–412. 19248407

[B17] Erdfelder E. Faul F. Buchner A. (1996). Gpower: A general power analysis program. Behav. Res. Methods, Instrum. Comput. 28 (1), 1–11. 10.3758/BF03203630

[B18] Fahmy S. F. Sabri N. A. El Hamamsy M. H. El Sawi M. Zaki O. K. (2016). Vitamin D intake and sun exposure in autistic children. Int. J. Pharm. Sci. Res. 7 (3), 1043–1049. 10.13040/IJPSR.0975-8232.7(3).1043-49

[B19] Gandhi R. T. Lynch J. B. del Rio C. (2020). Mild or moderate covid-19. N. Engl. J. Med. 383 (18), 1757–1766. 10.1056/NEJMCP2009249 32329974

[B20] Giménez V. M. M. Sanz R. L. Marón F. J. M. Ferder L. Manucha W. (2020). Vitamin D-RAAS connection: An integrative standpoint into cardiovascular and neuroinflammatory disorders. Curr. Protein Pept. Sci. 21 (10), 948–954. 10.2174/1389203721666200606220719 32504501

[B21] Giordo R. Zinellu A. Eid A. H. Pintus G. (2021). Therapeutic potential of resveratrol in COVID-19-associated hemostatic disorders. Molecules 26 (4), 856. 10.3390/molecules26040856 33562030 PMC7915700

[B22] Gorman S. Tan D. H. W. Lambert M. J. M. Scott N. M. Judge M. A. Hart P. H. (2012). Vitamin D3 deficiency enhances allergen-induced lymphocyte responses in a mouse model of allergic airway disease. Pediatr. Allergy Immunol. 23 (1), 83–87. 10.1111/J.1399-3038.2011.01146.X 22283404

[B23] Greiller C. L. Martineau A. R. (2015). Modulation of the immune response to respiratory viruses by vitamin D. Nutrients 7, 4240–4270. 10.3390/nu7064240 26035247 PMC4488782

[B24] Haddadi M. Pour M. (2022). IRCT approved: International and national patent: Herbal medicine for the treatment of COVID-19/PCT: WO2022009236A1/139950140003003167. Nigeria.

[B25] Hannan M. A. Rahman M. A. Sohag A. A. M. Uddin M. J. Dash R. Sikder M. H. (2021). Black cumin (nigella sativa L.): A comprehensive review on phytochemistry, health benefits, molecular pharmacology, and safety. Nutrients 13 (6), 1784. 10.3390/nu13061784 34073784 PMC8225153

[B26] Imran M. Khan S. A. Alshammari M. K. Alkhaldi S. M. Alshammari F. N. Kamal M. (2022). Nigella sativa L. and COVID-19: A glance at the anti-COVID-19 chemical constituents, clinical trials, inventions, and patent literature. Molecules 27, 2750. 10.3390/molecules27092750 35566101 PMC9105261

[B27] Jalal Z. Bakour M. Lyoussi B. (2021). Medicinal plants and zinc: Impact on COVID-19 pandemic. Sci. World J. 2021, 9632034. 10.1155/2021/9632034 PMC8483924 34602868

[B28] Kenneth Weir E. Thenappan T. Bhargava M. Chen Y. (2020). Does vitamin D deficiency increase the severity of COVID-19? Clin. Med. 20 (4), E107–E108. 10.7861/CLINMED.2020-0301 PMC7385774 32503801

[B29] Khazdair M. R. Ghafari S. Sadeghi M. (2021). Possible therapeutic effects of *Nigella sativa* and its thymoquinone on COVID-19. Pharm. Biol. 59 (1), 696–703. 10.1080/13880209.2021.1931353 34110959 PMC8204995

[B30] Kim T. H. Jeon S. R. Kang J. W. Kwon S. (2022). Complementary and alternative medicine for long COVID: Scoping review and bibliometric analysis. Evid. Based. Complement. Altern. Med. 2022, 7303393. 10.1155/2022/7303393 PMC9371860 35966751

[B31] Koshak A. E. Koshak E. A. Mobeireek A. F. Badawi M. A. Wali S. O. Malibary H. M. (2021). Nigella sativa for the treatment of COVID-19: An open-label randomized controlled clinical trial. Complementary Ther. Med. 61, 102769. 10.1016/J.CTIM.2021.102769 PMC8364675 34407441

[B32] Lai C. C. Shih T. P. Ko W. C. Tang H. J. Hsueh P. R. (2020). Severe acute respiratory syndrome coronavirus 2 (SARS-CoV-2) and coronavirus disease-2019 (COVID-19): The epidemic and the challenges. Int. J. Antimicrob. Agents 55 (3), 105924. 10.1016/J.IJANTIMICAG.2020.105924 32081636 PMC7127800

[B33] Leaf D. E. Ginde A. A. (2021). Vitamin D3 to treat COVID-19: Different disease, same answer. JAMA 325 (11), 1047–1048. 10.1001/JAMA.2020.26850 33595641 PMC8412177

[B34] Mahdavi R. Namazi N. Alizadeh M. Farajnia S. (2015). Effects of nigella sativa oil with a low-calorie diet on cardiometabolic risk factors in obese women: A randomized controlled clinical trial. Food Funct. 6 (6), 2041–2048. 10.1039/c5fo00316d 26029855

[B35] Malek Mahdavi A. (2020). A brief review of interplay between vitamin D and angiotensin‐converting enzyme 2: Implications for a potential treatment for COVID‐19. Rev. Med. Virol. 30 (5), e2119. 10.1002/RMV.2119 32584474 PMC7362103

[B70] Martineau A. R. Forouhi N. G. (2020). Vitamin D for COVID-19: A case to answer? Lancet Diabetes Endocrinol. 8 (9), 735–736. 10.1016/S2213-8587(20)30268-0 32758429 PMC7398646

[B36] Masoud H. Elassal G. Hassany M. Shawky A. Hakim M. Zaky S. (2020). Management protocol for COVID-19 patients MoHP protocol for COVID19 November 2020. Cairo Egypt: Ministry of health and population.

[B37] Meltzer D. O. Best T. J. Zhang H. Vokes T. Arora V. Solway J. (2020). Association of vitamin D status and other clinical characteristics with COVID-19 test results. JAMA Netw. Open 3 (9), e2019722. 10.1001/JAMANETWORKOPEN.2020.19722 32880651 PMC7489852

[B71] Mitchell F. (2020). Vitamin-D and COVID-19: Do deficient risk a poorer outcome? Lancet Diabetes Endocrinol. 8 (7), 570. 10.1016/S2213-8587(20)30183-2 32445630 PMC7239633

[B38] Muhammad T. (2021). SPECIFIC AIMS Fitra30 COVID-19 protocol. Cairo Egypt: Ministry of health and population.

[B39] Murai I. H. Fernandes A. L. Sales L. P. Pinto A. J. Goessler K. F. Duran C. S. C. (2021). Effect of a single high dose of vitamin D3 on hospital length of stay in patients with moderate to severe COVID-19: A randomized clinical trial. JAMA 325 (11), 1053–1060. 10.1001/JAMA.2020.26848 33595634 PMC7890452

[B40] Niranjan V. Setlur A. S. Karunakaran C. Uttarkar A. Kumar K. M. Skariyachan S. (2022). Scope of repurposed drugs against the potential targets of the latest variants of SARS-CoV-2. Struct. Chem. 33 (5), 1585–1608. 10.1007/s11224-022-02020-z 35938064 PMC9346052

[B41] Nugraha R. V. Ridwansyah H. Ghozali M. Khairani A. F. Atik N. (2020). Traditional herbal medicine candidates as complementary treatments for COVID-19: A review of their mechanisms, pros and cons. Evidence-based Complementary Altern. Med. 2020, 12. 10.1155/2020/2560645 PMC7569437 33101440

[B42] Phu H. T. Thuan D. T. B. Nguyen T. H. D. Posadino A. M. Eid A. H. Pintus G. (2020). Herbal medicine for slowing aging and aging-associated conditions: Efficacy, mechanisms and safety. Curr. Vasc. Pharmacol. 18 (4), 369–393. 10.2174/1570161117666190715121939 31418664

[B43] Ramazani E. Emami S. A. Tayarani-Najaran N. Sahebkar A. Tayarani-Najaran Z. (2021). Antiviral plants in view of avicenna's the canon of medicine and modern medicine against common cold. Adv. Exp. Med. Biol. 1328, 99–121. 10.1007/978-3-030-73234-9_7 34981473

[B44] Raslan M. Ms E. Ar S. Sabri N. A. (2022a). Microbiota in the era of COVID-19. Correlation and benefits. Act. Scie. Pharma. 6 (7), 22–29. 10.31080/ASPS.2022.06.0883

[B45] Raslan M. Ms E. Ar S. Sabri N. A. (2022b). The significance of COVID-19 vaccine booster dose. A comparative review. Act. Scie. Pharma. 6 (5), 03–07. 10.31080/asps.2022.06.0872

[B46] Rastogi A. Bhansali A. Khare N. Suri V. Yaddanapudi N. Sachdeva N. (2022). Short term, high-dose vitamin D supplementation for COVID-19 disease: A randomised, placebo-controlled, study (SHADE study). Postgrad. Med. J. 98 (1156), 87–90. 10.1136/POSTGRADMEDJ-2020-139065 33184146

[B47] Rostami S. Gharibi S. Yaghoobi H. Nokhodian Z. Shoaei P. Bahrami A. A. (2022). Herbal medicines as potential inhibitors of SARS-CoV-2 infection. Curr. Pharm. Des. 2, 2375–2386. 10.2174/1381612828666220802121014 35927924

[B48] Safdari M. R. Shakeri F. Mohammadi A. Bibak B. Alesheikh P. Jamialahmadi T. (2021). Role of herbal medicines in the management of brain injury. Adv. Exp. Med. Biol. 1328, 287–305. 10.1007/978-3-030-73234-9_19 34981485

[B49] Sen S. Singh B. Biswas G. (2022). Corticosteroids: A boon or bane for COVID-19 patients? Steroids 24, 109102. 10.1016/j.steroids.2022.109102 PMC9400384 36029810

[B50] Shaito A. Thuan D. T. B. Phu H. T. Nguyen T. H. D. Hasan H. Halabi S. (2020). Herbal medicine for cardiovascular diseases: Efficacy, mechanisms, and safety. Front. Pharmacol. 11, 422. 10.3389/fphar.2020.00422 32317975 PMC7155419

[B51] Shirvani H. Rostamkhani F. Arabzadeh E. Mohammadi F. Mohammadi F. (2021). Potential role of *Nigella sativa* supplementation with physical activity in prophylaxis and treatment of COVID-19: A contemporary review. Sport Sci. Health 17 (4), 849–854. 10.1007/s11332-021-00787-y 34093778 PMC8162633

[B52] Stroehlein J. K. Wallqvist J. Iannizzi C. Mikolajewska A. Metzendorf M. I. Benstoem C. (2021). Vitamin D supplementation for the treatment of COVID-19: A living systematic review. Cochrane Database Syst. Rev. 5 (5), CD015043. 10.1002/14651858.CD015043 34029377 PMC8406457

[B53] Ullah S. Munir B. Al-Sehemi A. G. Muhammad S. Haq I. U. Aziz A. (2022). Identification of phytochemical inhibitors of SARS-CoV-2 protease 3CLpro from selected medicinal plants as per molecular docking, bond energies and amino acid binding energies. Saudi J. Biol. Sci. 29 (6), 103274. 10.1016/j.sjbs.2022.03.024 35345871 PMC8944115

[B54] Wehbe Z. Hammoud S. Soudani N. Zaraket H. El-Yazbi A. Eid A. H. (2020). Molecular insights into SARS COV-2 interaction with cardiovascular disease: Role of RAAS and MAPK signaling. Front. Pharmacol. 3 (11), 836. 10.3389/fphar.2020.00836 PMC7283382 32581799

[B55] Wehbe Z. Wehbe M. Iratni R. Pintus G. Zaraket H. Yassine H. M. (2021). Repurposing ivermectin for COVID-19: Molecular aspects and therapeutic possibilities. Front. Immunol. 30 (12), 663586. 10.3389/fimmu.2021.663586 PMC8043070 33859652

[B56] World Medical Association (2013). World Medical Association declaration of Helsinki: Ethical principles for medical research involving human subjects. JAMA - J. Am. Med. Assoc. 310 (20), 2191–2194. 10.1001/jama.2013.281053 24141714

[B57] Yimer E. M. Tuem K. B. Karim A. Ur-Rehman N. Anwar F. (2019). Nigella sativa L. (Black cumin): A promising natural remedy for wide range of illnesses. Evid. Based. Complement. Altern. Med. 2019, 1528635. 10.1155/2019/1528635 PMC6535880 31214267

[B58] Younis N. K. Zareef R. O. Al Hassan S. N. Bitar F. Eid A. H. Arabi M. (2020). Hydroxychloroquine in COVID-19 patients: Pros and cons. Front. Pharmacol. 19 (11), 597985. 10.3389/fphar.2020.597985 PMC7751757 33364965

[B59] Zahedipour F. Hosseini S. A. Henney N. C. Barreto G. E. Sahebkar A. (2022). Phytochemicals as inhibitors of tumor necrosis factor alpha and neuroinflammatory responses in neurodegenerative diseases. Neural Regen. Res. 17 (8), 1675–1684. 10.4103/1673-5374.332128 35017414 PMC8820712

